# Perceptual Incongruence Influences Bistability and Cortical Activation

**DOI:** 10.1371/journal.pone.0005056

**Published:** 2009-03-31

**Authors:** Gijs Joost Brouwer, Frank Tong, Peter Hagoort, Raymond van Ee

**Affiliations:** 1 Department of Physics, Helmholtz Institute, Utrecht University, Utrecht, The Netherlands; 2 Department of Psychology, Vanderbilt University, Nashville, Tennessee, United States of America; 3 F.C. Donders Center for Cognitive Neuroimaging, Nijmegen, The Netherlands; University of Minnesota, United States of America

## Abstract

We employed a parametric psychophysical design in combination with functional imaging to examine the influence of metric changes in perceptual incongruence on perceptual alternation rates and cortical responses. Subjects viewed a bistable stimulus defined by incongruent depth cues; bistability resulted from incongruence between binocular disparity and monocular perspective cues that specify different slants (slant rivalry). Psychophysical results revealed that perceptual alternation rates were positively correlated with the degree of perceived incongruence. Functional imaging revealed systematic increases in activity that paralleled the psychophysical results within anterior intraparietal sulcus, prior to the onset of perceptual alternations. We suggest that this cortical activity predicts the frequency of subsequent alternations, implying a putative causal role for these areas in initiating bistable perception. In contrast, areas implicated in form and depth processing (LOC and V3A) were sensitive to the degree of slant, but failed to show increases in activity when these cues were in conflict.

## Introduction

Bistability is a powerful paradigm to investigate perception and its underlying neural mechanisms, a phenomenon during which perception alternates between two interpretations of a single, constant stimulus [1]. A defining characteristic of bistability is the rate at which perception alternates. The frequency of perceptual alternations is influenced by a number of factors including visual attention [2], [3], mood disorders [4] and neurological disorders [5]. Although there has been considerable investigation into neural correlates of bistable perception [6]–[15] little is known about the neural mechanisms representing the degree of perceptual incongruence between conflicting inputs.

Bistable perception is thought to result from neural competition between conflicting perceptual representations. This suggests that if the level of incongruence between two competing representations increases, neural competition increases, possibly resulting in more frequent alternations.

**Figure 1:**
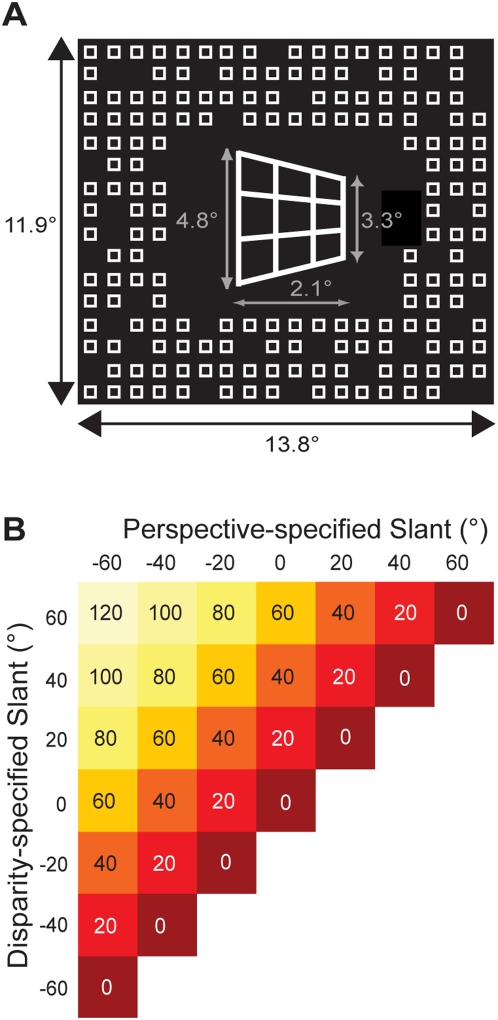
Stimulus design and used configurations. (A) To produce slant rivalry between the perceived surface slant dominated by perspective and the slant dominated by disparity, we independently varied perspective- and disparity-defined slants. Due to foreshortening, slant-rivalry stimuli with different perspective settings have slightly different sizes (average sizes are shown). Stimuli were presented within an aperture of a surrounding pattern (13.8° by 11.9°) consisting of small squares (0.5° by 0.5°) providing a zero-slant reference that prevented depth contrast illusions. (B) 49 different slant-rivalry stimuli were created by varying disparity-defined and perspective-defined slant independently between −60° and 60°, in step of 20°. For the psychophysical experiments, we used 28 out of 49 possible stimuli, corresponding roughly to half of the symmetric stimulus space. For the functional imaging experiments, we included four high incongruence stimuli (80°, 100°, 100° and 120° of incongruence) of the other half of the stimulus space while removing two low incongruent (incongruence of 20°) from the set that was used for the psychophysical experiments. This was done to balance the number of high incongruence stimulus versus the low incongruence stimuli.

To test this, we use slant rivalry [16] in which bistable perception originates from conflict between two slant-defining cues, binocular disparity and monocular perspective, resulting in perceptual alternations between a perspective-dominated and disparity-dominated percept, **Fig. 1a**. This stimulus is well suited because incongruence can be metrically altered by independently changing perspective and disparity-defined slants. Previously, we found that the temporal dynamics of slant rivalry are similar to other examples of perceptual bistability [17], that slant-defining signals adapt independently [18], and we used functional imaging to identify cortical activation correlating with the perception of stereoscopic slant [19].

Here, we investigated whether the degree of perceptual conflict or incongruence has a systematic effect on the dynamics of bistable perception and the strength of cortical responses. If so, it would demonstrate that the brain takes into account the relative difference between interpretations in its attempt to reconcile these into a coherent, stable perceptual experience. In the psychophysical experiments, we used a subset of stimuli from a slant-rivalry stimulus space, defined by independently varying disparity and perspective-defined slants (**Fig. 1b**) to determine the effect of incongruence on alternation rates.

Using fMRI, we investigated the neural correlate of perceptual conflict, which with prolonged viewing, leads to more frequent perceptual alternations. Alternations have been shown to activate areas of extrastriate, parietal and prefrontal cortex [8]–[11], [19]. Here, we specifically investigated the cortical response to the level of incongruence. To isolate these responses, stimuli were presented briefly to minimize the likelihood of alternations occurring.

We predicted that brain areas sensitive to perceptual conflict should show differential activity as a function of incongruence, independent of the level of disparity- and perspective-defined slants. By presenting stimuli too briefly to allow for perceptual alternations, we were able to isolate changes in activity as a function of incongruence independent of activity associated with perceptual alternations.

Using this novel parametric approach, we show that alternation rates are accurately predicted by the incongruence between perceptual interpretations. We further demonstrate that the anterior part of the intraparietal sulcus shows systematic increases in activity as a function of perceptual incongruence.

## Methods

### Subjects

Ten subjects participated in this study. Of these subjects, seven were available for extensive psychophysical testing performed outside of the MRI scanner. The remaining three participated only in the imaging experiment. Subjects had normal or corrected-to-normal vision. Subjects' stereovision was tested using a stereoanomaly test that capitalized on their ability to distinguish between crossed and uncrossed disparities of magnitudes between −1 to 1 deg, without the possibility that eye movements could mask a deficiency [20]. Subjects' stereovision differed along a continuous spectrum. For example, on one side of the spectrum subject LD was excellent at distinguishing the signs and magnitudes of both the crossed and the uncrossed disparities. On the other side of the spectrum MD was just above chance in distinguishing the signs and magnitudes of disparity and RV was entirely unable to do so. We included RV as a subject as he provides converging evidence for the relationship between perceptual incongruence and cortical activation: due to his poor stereovision, the subject showed no effect of incongruence on alternation rates, as well as showing no change in cortical activation in response to changes in incongruence. This makes it unlikely that the observed pattern of activation found in other subjects was due to attentional confounds, unrelated to the processing of depth information and perceptual conflict. Functional imaging procedures were approved by the FC Donders Centre for Cognitive NeuroImaging. Informed written consent was obtained prior to scanning.

### Visual Stimuli

#### Slant-Rivalry Stimuli

Using the OpenGL graphics engine, a wire frame rectangle (consisting of four vertical and four horizontal lines) was rotated about the vertical axis to create the trapezoidal shape (perspective-defined surface slant; **Fig. 1a**). We created a disparity gradient (disparity-defined surface slant) by horizontally compressing one eye's half-image and magnifying the other eye's half-image. We utilized a conventional red-green anaglyphic technique to present the stimuli stereoscopically (see for demonstrations http://www.phys.uu.nl/~vanee). Photometric measurements demonstrated that only minute amounts of the green and the red light leaked through the red (0.4%) and the green (0.2%) filter, respectively. We independently varied the disparity- and perspective-defined slant in steps of 20° between −60*°* and 60*°*, creating a stimulus set of 49 slant-rivalry stimuli (see **Fig. 1b**). The level of incongruence between slant-specifying cues was defined as the absolute difference in angle between cues, relative to the fronto-parallel plane. Average stimulus-width was 2.1°. Average height of the stimulus was 4.8° (left side) and 3.3° (right side). The background (13.8° by 11.9°) consisted of an array of small squares (0.5° by 0.5°) to facilitate stereofusion; 80% of the squares in this array were shown to prevent fixation in the wrong depth plane (i.e. wallpaper effect). The slant rivalry display was shown within an aperture in the background at fixation.

For the slant-rivalry psychophysical experiments, we used 28 out of the 49 possible stimuli, corresponding roughly to half of the symmetric stimulus space. For the slant-estimation psychophysical experiments, we used all 49 possible stimuli. For the functional imaging experiments, we included four high incongruence stimuli (80, 100, 100*°* and 120*°* of incongruence respectively) of the other half of the stimulus space while removing two low incongruent stimuli (incongruence of 20*°*) from the set that was used for the psychophysical experiments. This was done to balance the number of high and low incongruence stimuli (in the full stimulus set, 120*°* of incongruence occurred only twice, while 0*°* of incongruence occurred 7 times), see **Table 1**. For the psychophysical experiments, stimuli were presented using a LaCie monitor (resolution 1600×1200 pixels) with subjects seated at 52 cm distance from the screen. During functional imaging, stimuli were presented using an EIKI projector (LC-X986, resolution 800×600 pixels) onto a transparent screen positioned at the rear end of the MR scanner. Subjects viewed these through a mirror attached to the head coil. Distance to the screen via the mirror was 80 cm. Red and green filters were attached to MR-suited glasses for viewing of our stereoscopic stimuli.

**Table 1:** Summary of the slant-rivalry stimuli used in the imaging experiments.

Perspective	Disparity	Incongruence	
60	−60	120	**Slant 60, Incongruent**
60	−40	100	
60	−20	80	
60	0	60	
60	20	40	
60	60	0	**Slant 60, Congruent**
40	−60	100	
40	−40	80	**Slant 40, Incongruent**
40	−20	60	
40	0	40	
40	40	0	**Slant 40, Congruent**
20	−60	80	
20	−40	60	
20	−20	40	**Slant 20, Incongruent**
20	0	20	
20	20	0	**Slant 20, Congruent**
0	−60	60	
0	−40	40	
0	−20	20	
0	0	0	**Slant 0**
−20	−40	20	
−20	−20	0	**Slant 20, Congruent**
−20	20	40	**Slant 20, Incongruent**
−40	−60	20	
−40	−40	0	**Slant 40, Congruent**
−40	40	80	**Slant 40, Incongruent**
−40	60	100	
−60	−60	0	**Slant 60, Congruent**
−60	40	100	
−60	60	120	**Slant 60, Incongruent**

The color coding of the columns indicates the subset of stimuli used for the congruent/incongruent analysis described in **Figure 5**. These particular stimuli represent cases in which there equal slant information in both perspective and disparity-defined cues, either opposite (bright red) or equal in slant.

#### Polar Retinotopic Mapping / MT+ and LOC Localization Stimuli

To delineate borders between visual areas, we relied on retinotopic mapping data collected in a prior scanning session. Polar retinotopic mapping was done using methods described in detail previously [19], [21]–[24]. We used two rotating wedges to map the visual field based on previously described methods [14]. Contained within these wedges was a contrast-reversing checkerboard pattern that flickered at 8 Hz. We used this method of two wedges since it produces a more stable stimulus, reducing unwanted eye movements. For mapping of MT+, we used a block design consisting of epochs of randomly located stationary dots, interleaved with epochs of randomly located dots moving away from the center (outward radial motion at a velocity of 3°/s). For mapping of LOC [25]–[27], we used a block design consisting of epochs of objects (faces, houses, scenery and man-made objects) or scrambled versions of the same images, described in detail in [27].

### Procedure

#### Psychophysical Experiments: Slant Estimation

During the slant-estimation psychophysical experiments, subjects viewed stimuli for a duration of 3 seconds, after which they estimated both the perceived slant that was dominated by the disparity cue and the perceived slant that was dominated by the perspective cue. They did so using a schematic top view of the stimulus, adjusting the orientation of two lines representing the slants [16] as seen from above. A sensible objection to this metrical slant-estimation method is that it is hard to interpret the data because a slant angle that is estimated at 35 deg in one trial might look like 40 deg in another trial. Previous work has demonstrated, however, that subjects have a relatively constant internal reference and that they do not regard this task as difficult. This estimation method has been used previously for real planes and when subjects wore distorting lenses [62]. In addition, a similar metrical depth estimation method was successfully used for volumetric stimuli [63]. All 49 possible stimuli were presented 4 times.

#### Psychophysical Experiments: Slant Rivalry

During the slant-rivalry psychophysical experiments, subjects viewed stimuli for a duration of 210 seconds. A total of 28 different stimuli were presented twice in each of a total of four sessions. Subjects were required to maintain fixation at the center of the stimulus (which they can easily do when viewing a slant rivalry stimulus [17], [28]), using two buttons to indicate the predominance of either percept. Subjects were instructed to report which side (left or right side) of the stimulus was perceived as closer, relative to the other side. As an additional control, after each trial, subjects again estimated both the perceived slant that was dominated by the disparity cue and the perceived slant that was dominated by the perspective cue.

#### Slant Rivalry Functional Imaging Experiments

During the functional imaging experiments, subjects viewed a total of 30 different stimuli (see **Table 1** and see **Fig. 1**), each presented twice per run using a jittered event-related design. Stimuli were presented for 1 second, interleaved with blank displays containing a yellow fixation dot, lasting between 3 and 7 seconds. These interstimulus intervals were chosen randomly such that the orthogonality of the resulting design matrix was maximal (average correlation <0.1 between predictors). Subjects were instructed to keep fixation either on the fixation dot (during fixation epochs) or on the center of the stimulus. Runs (6 to 7 per subject) lasted 720 seconds.

#### Retinotopic mapping/localization of MT+ and LOC

Retinotopic mapping and functional mapping of area MT+ was performed using methods identical to those described previously [19], [21]. All subjects performed three polar mapping runs, consisting of 10 cycles (full hemifield rotation of two wedges), lasting a total of 456 seconds. In addition, subjects performed one run of MT+ localization and one run of LOC localization. MT+ localization runs consisted of six 16-second epochs of stationary dots and six 16-second epochs of outward radial motion, interleaved with 16-second fixation rest periods. During LOC localization runs, subjects viewed 16-second epochs of various image types (*see above*), interleaved with 16 second epochs containing a blank fixation screen. Within image epochs, a total of 25 randomly chosen stimuli were shown for 500 msec, interleaved with 160 msec blanks. Contrasting epochs containing objects (houses, faces, scenery, man-made objects) with scrambled versions of the same objects localizes area LOC [27].

### Magnetic Resonance Imaging

All images were acquired using a 3 Tesla Siemens TRIO with exception of a high-resolution T1 anatomical scan acquired using a 1.5 Tesla Siemens Sonata. Scanners were located at the FC Donders Centre for Cognitive NeuroImaging, Nijmegen, The Netherlands. We used a 1-mm resolution 3D-MPRAGE (*optimized contrast between gray and white matter*) for high-resolution anatomical scans. All functional images were collected using Echo Planar Imaging (EPI). For runs with bistable stimuli we used 25 horizontal slices (TR = 2000 ms, TE = 30; 64×64 matrix; voxel size 3.5 mm^3^). For retinotopic mapping we used 25 horizontal slices (TR = 3000 ms, TE = 30; 64×64 matrix, voxel size 3×3×3 mm).

### Cortical Flattening and Area Border Delineation

The cortical sheets of the individual subjects were reconstructed as polygon meshes based on the high-resolution T1 scans. The white-gray matter boundary was segmented, reconstructed, smoothed, inflated and flattened [29]. Area border delineation using the polar retinotopic mapping was done using methods previously described [22]–[24], [30], [31]. Using the correlation between wedge position and neural activity, borders were identified on the basis of field-sign alternations and areas were drawn in on the flattened sheet manually.

### Functional MR Data Analysis

We used BrainVoyagerQX (*BrainInnovation, the Netherlands*) and Matlab (*Mathworks*) for all functional data analysis as well as for the creation of flattened cortical representations. Before analysis, we removed the first three volumes of every scan. All remaining functional images were subjected to a number of preprocessing steps: 1) motion correction, 2) slice timing correction 3) linear trend removal using a high pass filter and 4) transformation of the functional data into Talairach coordinate space [32]. We convolved the duration of the stimuli (1 second) with a standard hemodynamic model of BOLD activation [33] and estimated the changes in BOLD signal given a particular stimulus using the general linear model or GLM [34].

To map areas showing an effect of incongruence on activation, we used a random-effects GLM group analysis (*threshold: p<0.001*), contrasting the stimuli with the three highest incongruencies (80*°*, 100*°*, 120*°*) against the stimuli with the three lowest incongruencies (0*°*, 20*°* and 40*°*). The data from the resulting clusters of activation for which this contrast reached statistical significance was then further analyzed using linear regression to determine the correlation between stimulus measures and cortical activation (level of disparity-defined slant, level of perspective-defined slant, level of incongruence).

For a region-of-interest-based analysis of the visual areas (V1, V2, V3, V3A, VP, V4V and MT+), we used only the voxels that were activated significantly (p<0.001, corrected) by the extent of the stimulus.

In an additional analysis, we compared normalized BOLD signal changes of stimuli with identical and equal levels of disparity and perspective-defined slants (*e.g. 60° disparity-defined slant / 60° perspective-defined slant, no incongruence*) with stimuli of identical but opposite levels of disparity and perspective-defined slants (*e.g. 60°/−60°, incongruence 120°*) within these various regions of interest. Incongruence remains at 0*°* if one increases the level of both slant-cues (from 0*°* to 60*°*, in steps of 20*°*) according to a common sign or direction. By contrast, increasing the level of both slant cues while making them opposite in sign does increase incongruence from 0*°* (*0/0*) to 120*°* (*e.g. 60°/−60°*). At each level of cue-defined slant, we compared the activation associated with same sign stimuli (no incongruence) with those of opposite sign (incongruent) using a student t-test statistic. Event-related averages were created by calculating average time courses using a fixed time window (*−4 to +18 sec*) that was centered on the time a particular stimulus was presented.

## Results

### Psychophysical results: Slant Estimates

**Figure 2:**
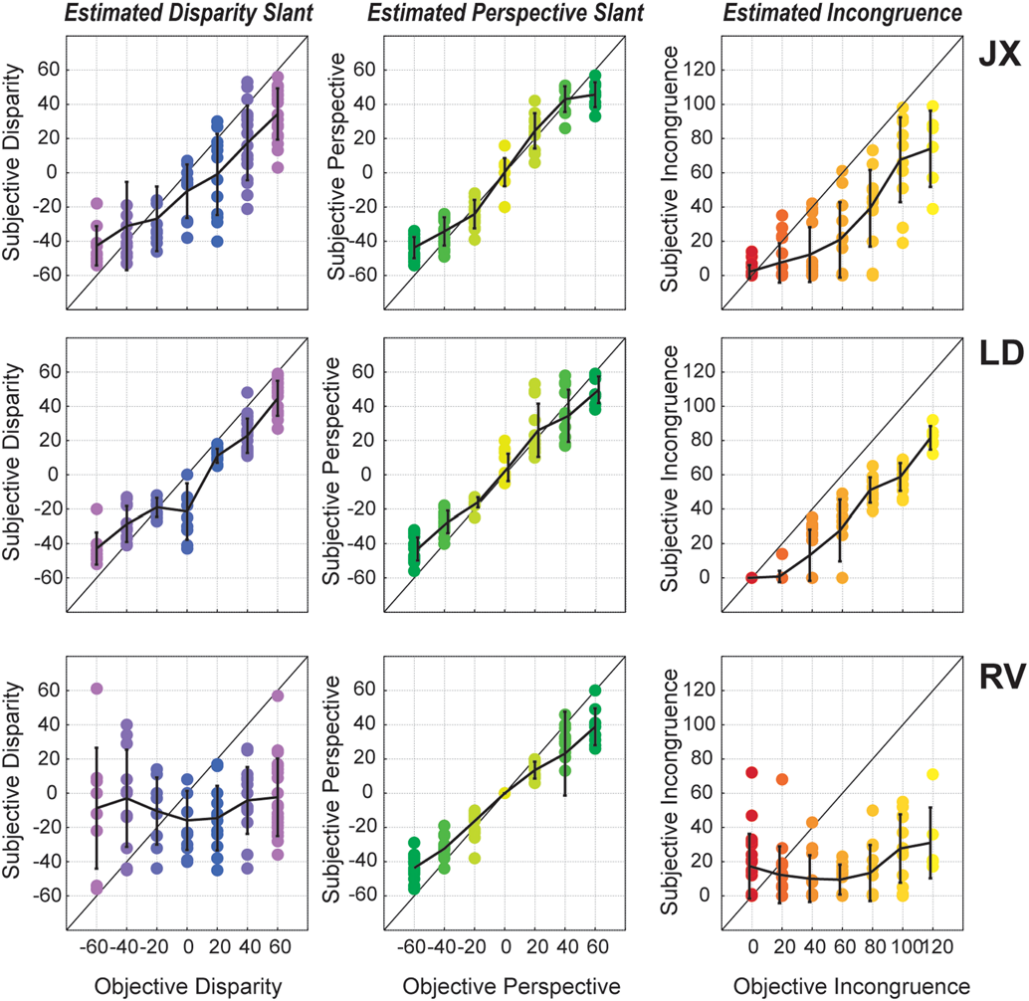
Slant estimates for three representative subjects. Subjects estimated both the perceived surface slant that was dominated by the disparity cue and the perceived slant that was dominated by the perspective cue. Each data point represents one single estimate. The abscissa reflects the slant defined by disparity (left) and perspective (middle). From these estimates, we calculated the subjective level of incongruence (right). For subjects JX and LD, estimates are reliable, although somewhat underestimated, resulting in a correlation between objective and subjective incongruence. For subject RV, who is deliberately included because his stereovision proved to be poor, estimation of the disparity-defined slant is poor, and as a result so is the correlation between objective and subjective incongruence. Errorbars denote SD.

In the first analysis, we investigated the relationship between objective measures of slant (actual presented slant) and subjective measures of slant (perceived slant). From this, we could derive an estimate of subjective incongruence, based on the perceived angular difference (in the 3D depth plane) between the disparity-defined and perspective-defined slant. Objective incongruence reflected the actual angular difference between these conflicting slant cues. In accordance with earlier findings, perceived slants were typically underestimated with regard to the slant information present in the stimulus [20], [28]. In addition, low levels of incongruence (*e.g. 20 or 40°*) were perceptually reconciled and perceived as being congruent: subjects typically reported perceiving an intermediate slant, indicating that the conflicting cues were integrated into a coherent percept, as was shown in greater detail in a previous study, suggesting a Bayesian estimator that reconciles available slant cues [35]. Slant estimates for three representative subjects (**JX**, **LD** and **RV**) are shown in **Fig. 2a**. For these three subjects, the correlation between presented and estimated perspective-defined slant was quite accurate (*middle column*), although the actual slant was underestimated. The correlation between presented and estimated disparity-defined slant was somewhat less reliable, and even quite low in one of the subjects (**RV**, *bottom row*). We deliberately included **RV** because of his poor stereovision. We argued that if this subject was poor in estimating slant on the basis of stereoscopic depth cues, the effect of incongruence (based upon the conflict between disparity and perspective) should be minimal or even absent. Indeed, while the estimated incongruence for subjects **JX** and **LD** corresponded well with the actual incongruence, the estimated incongruence of **RV** clearly does not. In accordance with this performance and his poor stereovision, this subject also showed a weak correlation between incongruence and alternation rates, as well as between incongruence and cortical activation (*see below*). **Table 2** summarizes the statistics of the correlation between presented and perceived disparity- and perspective-defined slants and between presented and perceived incongruence for all subjects (r-squared values derived from the fits). Finally, the reliability of slant estimates does not seem to depend on incongruence: estimation accuracies are similar for all levels of incongruence. Apparently, the conflict between slants does not interfere with the consistency of subjects' estimates of each slant component, although, at the same time, it can have profound effect on how the stimulus is perceived.

**Table 2:** Summary of the correlation between perceived and presented disparity- and perspective-defined slants (columns 1 and 2) and between perceived and presented incongruence (column 3).

	Perspective	Disparity	Incongruence
**JB**	0.91	0.86	0.79
**TK**	0.97	0.97	0.94
**LD**	0.90	0.85	0.87
**AK**	0.83	0.53	0.46
**MD**	0.77	0.57	0.40
**RV**	0.94	0.00*	0.04*
**GB**	0.97	0.89	0.86
**JX**	0.93	0.68	0.69
**DW**	0.93	0.52	0.70
**CK**	0.88	0.22	0.33
**WS**	0.93	0.72	0.21

With the exception of subject RV, all subjects show a significant correlations between presented and perceived measures of slant and incongruence. Values indicate R-squares derived from the fits between perceived and presented measures. Astrix indicate fits not reaching significance (p>0.05).

### Psychophysical results: Alternation rates

**Figure 3:**
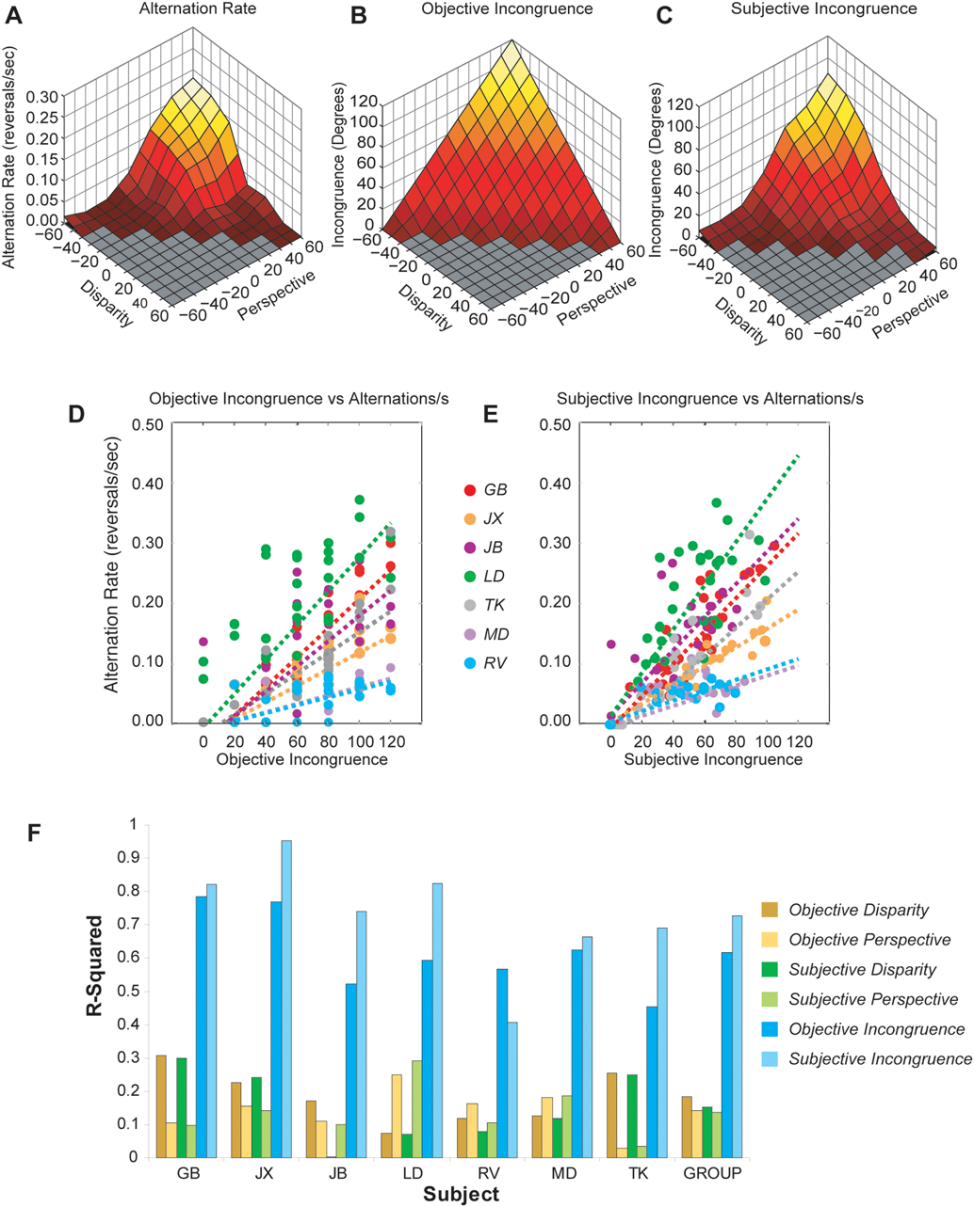
The correlation between alternation rates and subjective incongruence in all subjects. (A) The effect of independently varying the level of disparity-defined slant (x-axis) and perspective-defined slant (y-axis) on alternation rates (z-axis). Since the stimulus space formed by varying disparity and perspective is symmetrical, only half of the available stimuli were presented to the subjects. Color coding depicts the level of objective incongruence for each stimulus (dark red: low incongruence, yellow/white: high incongruence). Alternations rates increase with increased levels of incongruence. (B) The objective level of incongruence for all test stimuli (z-axis) as a function of independently varying disparity- (x-axis) and perspective-defined slant (y-axis). Objective levels of incongruence are computed as the absolute difference between the disparity- and perspective-defined slant of each stimulus. (C) The subjective level of incongruence, as estimated by subjects, with axes identical to (B). The increase in alternation rates depicted in (A) is better predicted by subjective incongruence (C) than by objective incongruence (B). (D) Trial-by-trial scatter plot, correlating objective incongruencies (abscissa) with alternation rates (ordinate) for all individual subjects (color-coded). This demonstrates that differences in alternation rates between subjects are observed, but that in all cases a positive correlation can be observed between incongruence and alternation rate. A similar observation can be made by observing the correlation between subjective incongruence and alternation rate (E). Summarizing all psychophysical results, (F) shows that of all measures (objective disparity, perspective, incongruence; subjective disparity, perspective, incongruence), subjective incongruence is the best predictor of subsequent alternation rates. The only exception is subject RV, in accordance with his poor stereovision.

A first novel finding was that alternation rates increase with higher incongruencies. **Fig. 3** shows group psychophysical data. Comparing the increase in alternation rate (**Fig. 3a**) with both objective (**Fig. 3b**) and subjective incongruence (**Fig. 3c**) of the presented stimuli reveals the latter to be more correlated with alternation rates than the former. The effect of incongruence on alternation rates can also be observed directly on a trial-by-trial basis (s*hown for objective incongruence in* **Fig. 3d** *and subjective incongruence in* **Fig. 3e**). Here, individual differences in alternation rate become apparent: the magnitude of the increase in alternation as a result of incongruence varies between subjects. Nevertheless, for all subjects an increase in alternation rate was observed for higher incongruencies. Although objective and subjective measures of incongruence are both predictive of alternation rates, subjective incongruence was more closely related to the alternation rate than the stimulus' objective incongruence. **Fig. 3f** summarizes the statistical results, showing that of all measures, subjective incongruence appears to be the best predictor of alternation rate, outperforming prediction of alternation rate on the basis of disparity and perspective cues (*both objective and subjective*) and objective incongruence. Statistical analyses of these results show that objective incongruence is a better predictor of alternation rate than both objective disparity-defined slants (*paired sample t-test*, *t_5_ = 9.52*, *p<0.01*) and objective perspective-defined slants (*paired sample t-test*, *t_5_ = 9.85*, *p<0.01*). In addition, subjective incongruence outperforms subjective disparity-defined slants (*paired sample t-test*, *t_5_ = 13.31*, *p<0.01*) as well as subjective perspective-defined slants (*paired sample t-test*, *t_5_ = 31.05*, *p<0.01*). The difference between subjective and objective incongruence failed to reach significance, due to the opposite effect observed in subject RV, for whom objective incongruence outperforms subjective incongruence as being the better predictor of alternation rates. Taken together, though, these results suggest that the mechanism underlying the temporal dynamics of slant rivalry is sensitive to the difference between interpretations of surface slant cues, rather than their absolute levels.

#### fMRI results: Activation of the anterior intraparietal sulcus correlates with incongruence

**Figure 4:**
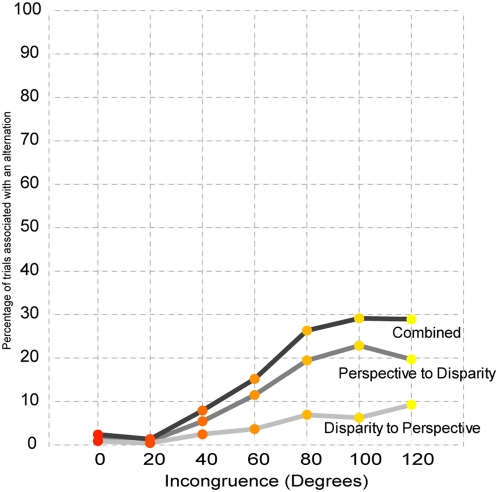
Percentage of trials in which a perceptual alternation occurred during the imaging experiments. Although stimuli were presented briefly (1-second), alternations were occasionally being reported. The likelihood of an alternation increased at higher levels of incongruence, mimicking the psychophysical results. In addition, the majority of these alternations (68%) involved switches from the perspective-dominated percept to the disparity-dominated percept.

To examine the influence of incongruence on cortical activation, we used functional magnetic resonance imaging (fMRI) while subjects viewed a subset of the slant rivalry stimuli, presented briefly for 1 second. These short durations allowed us to study the effect of incongruence on cortical activity prior to the onset of alternations. Even so, in some instances subjects reported alternations. The chance of an alternation occurring during the 1-second presentation increased as a function of incongruence, mimicking the psychophysical results. At the highest level of incongruence, alternations were experienced in less than 30% of all trials. In addition, most alternations (68%) were alternations from the perspective-dominated percept towards the disparity-dominated percept (see **Fig. 4**).

**Figure 5:**
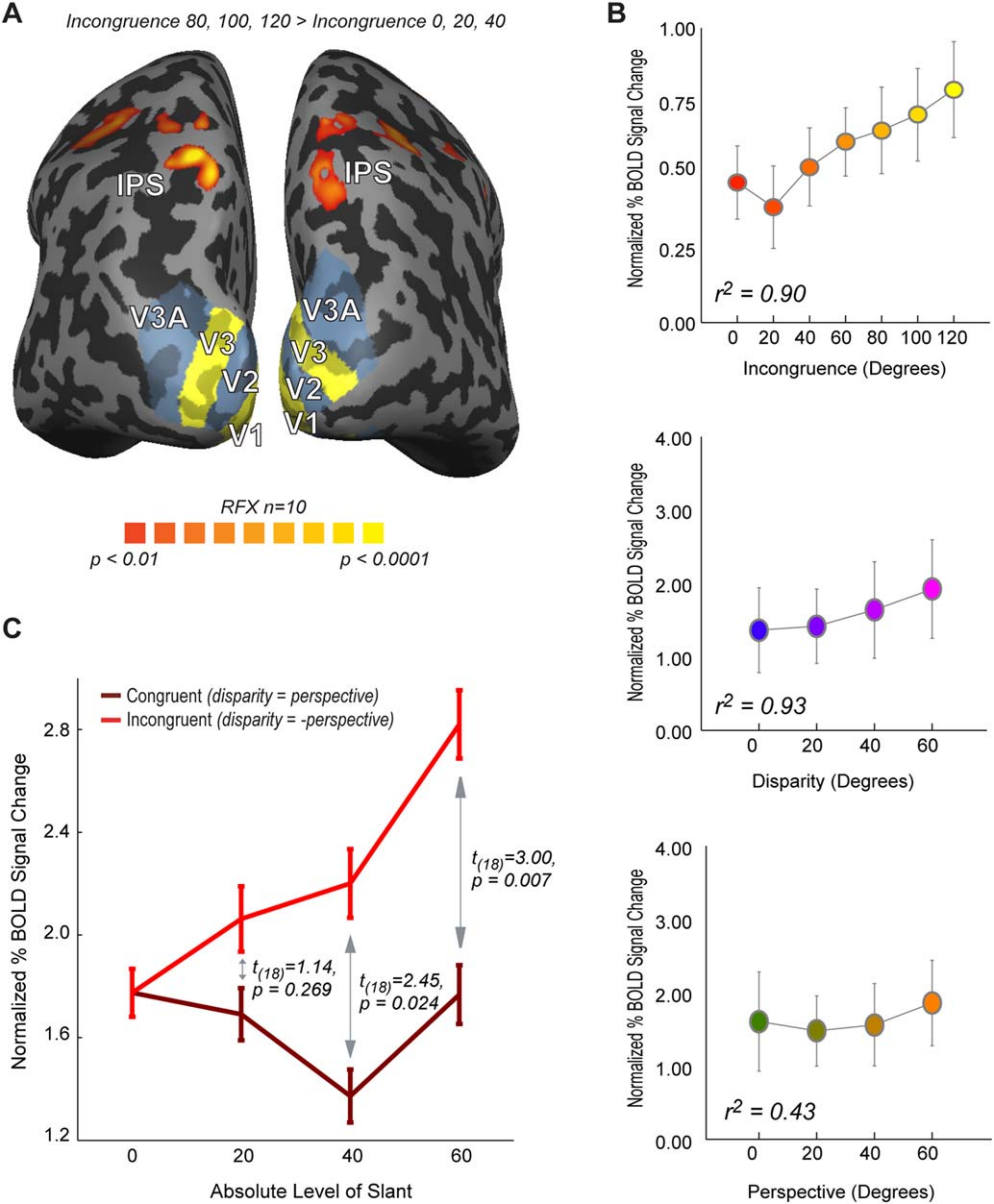
Imaging Results (A). Results of random-effects GLM analysis, contrasting the three highest levels of incongruence (*80*°,*100*° *and 120*°) against the three lowest levels of incongruence (*0*°, *20*° *and 40*°). Bilateral activation for this contrast was found along the anterior part of the intraparietal sulcus. (B) Changes in percentage BOLD signal as a function incongruence (*top*), disparity-defined slant (*middle*) and perspective-defined slant (*bottom*). (C) Comparison of the difference in evoked activation between stimuli containing equal (*no incongruence*) and opposite (*incongruence*) levels of disparity and perspective-defined slants. Increasing the level of disparity- and perspective-defined slants did not significantly increase the activation when these slants were kept identical (*e.g. perspective-defined slant: 60°*, *disparity-defined slant: 60°*, *dark red line*). If slant levels are increased in opposite directions, then this increase in incongruence is associated with increased cortical activation. Taken together, these data demonstrate that activation that the activation cluster within IPS reflects incongruence, independent of disparity- and perspective-defined slant. Error bars denote SEM.

In a first analysis, we identified the voxels that were more active for the three highest levels of incongruence (*80*, *100*, *120°*) versus the three lowest levels of incongruence (*0*, *20*, *40°*). This contrast (**Fig. 5a**) revealed a cluster of robust bilateral activation in the anterior part of the intraparietal sulcus (aIPS cluster, *random effects analysis*, *t_9_>5.88*, *p<0.0005*). The data taken from this cluster was used to examine the precise relationship between cortical activation and various stimulus manipulations of disparity-defined slant, perspective-defined slant and the incongruence between them. **Fig. 5b** plots the normalized BOLD signal changes for each level of incongruence (*top panel*), the level of disparity-defined slant (*middle panel*) and the level of perspective-defined slant (*bottom panel*), together with the results from a linear regression analysis. The activation within the aIPS cluster of activation correlates positively with all three measures. More importantly, activation correlates with the level of incongruence: higher levels of incongruence between perceptual states evoke a higher response within the intraparietal cluster of activation. One concern is that the activation also seems to correlate highly with the absolute level of disparity- and perspective-defined slant. As explained above, the level of incongruence of a stimulus is inevitably correlated with the level of both disparity and perspective-defined slants to some degree, as higher levels of slant are necessary to create higher levels of incongruence. It is therefore crucial to compare stimuli with different levels of incongruence with congruent stimuli containing identical levels of disparity- and perspective-defined slant. We therefore compared cortical responses to stimuli in which disparity- and perspective-defined slants specified identical but opposite levels of slant (*creating incongruence*, *e.g. 60° and −60°*) with those of equal slant (*no incongruence*, *e.g. 60° and 60°*). By comparing the difference in evoked activation between stimuli containing equal (*no incongruence*) and opposite (*incongruence*) levels of disparity- and perspective-defined slant, we can separate activation changes correlated with increased levels of incongruence from activation changes correlated with increased levels of slant. The results for this particular analysis are shown in **Fig. 5c**. Here, for our aIPS cluster of activation, increasing the level of disparity and perspective-defined slant does not lead to an increase in activation when these slants are in agreement (*dark red line*). If, however, slant levels increase in opposite directions, the effect of which is an increase in incongruence between slants (*bright red line*), we see an increase in associated cortical activation. At the group level, this effect does not reach significance for the comparison between incongruent and congruent slants of 20*°* (*t_18_ = 1.14*, *p = 0.269*), but the comparisons between incongruent and congruent slants of 40*°* and 60*°* show that incongruent slants are associated with higher signal changes than are congruent slants of the same magnitude (40*°*: *t_18_ = 2.45*, *p = 0.024*, 60*°*: *t_18_ = 3.00*, *p = 0.007*). Taken together, our data demonstrate that the activation in IPS is strongly correlated with incongruence, independent of disparity and perspective-defined slants.

One potential concern is that the increase in activation as a function of incongruence might be due to the confounding presence of alternations, as these were more likely to occur under conditions of high incongruence. Even though a relatively low percentage of trials were accompanied by a perceptual alternation (reaching a maximum of about 30 percent for trials with the highest levels of incongruence), we nevertheless removed these trials for an additional analysis. Analysis of only the non-alternation trials revealed the same pattern of results as was obtained from the entire data set, with statistically significant effects of comparable magnitude found in both cases.

**Figure 6:**
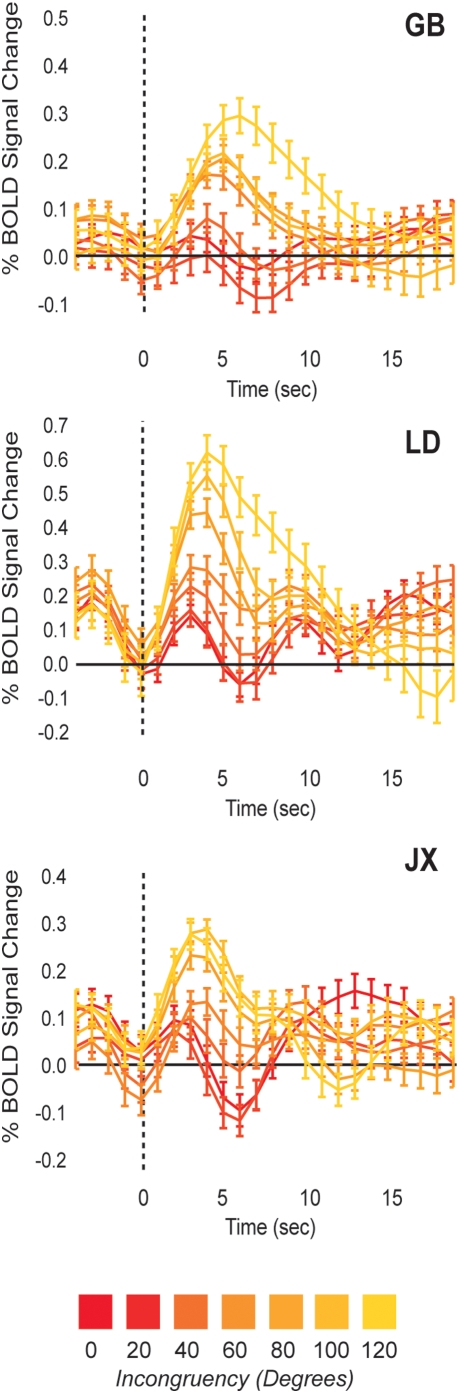
Event-related averages. Event-related averages of the activation evoked by different levels of incongruence for three representative subjects. The higher the level of incongruence, the higher the evoked BOLD signal changes in aIPS. Error bars denote SEM.

**Figure 7:**
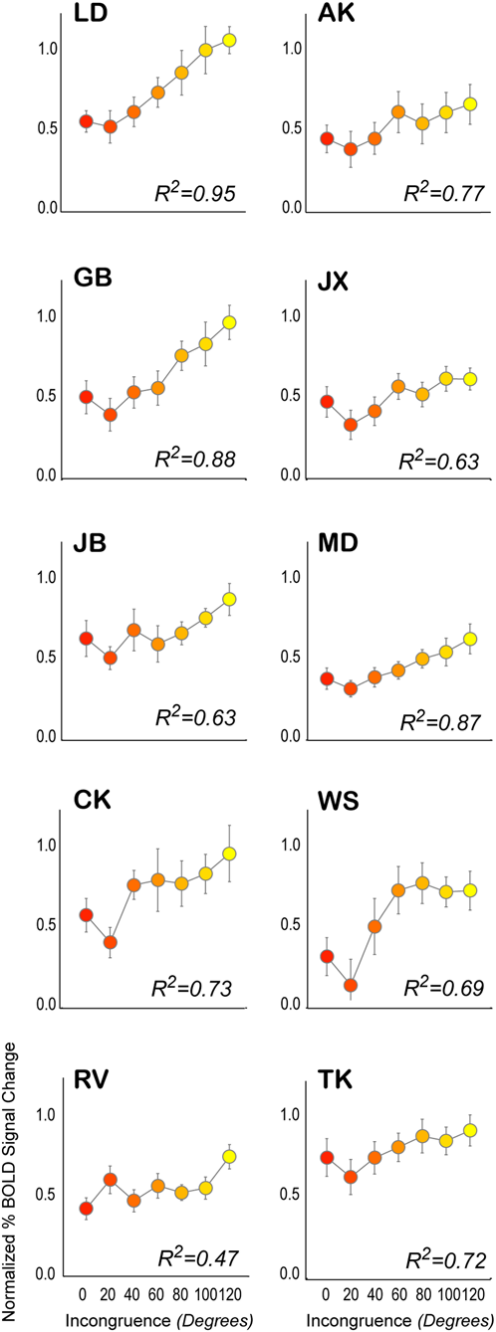
Correlations between incongruence and cortical activation for all individual subjects. Overall, all subjects show a significant increase in activation for higher incongruencies. However, for RV this increase is somewhat weak. This fits nicely with the psychophysical results: this subject was poor at estimating the level of disparity-defined slant (Fig. 2) and, as a result, the level of incongruence within a stimulus. In addition, the influence of incongruence on alternation rate was also low for this subject. Error bars denote SEM, insets show the statistical result of the linear regression between incongruence and normalized BOLD signal changes, expressed in the fit parameter R-squared.

The effect of increasing incongruence on cortical activation within aIPS can also be seen in plots showing event-related averages of BOLD signal changes (**Fig. 6**, *three representative subjects*). Furthermore, **Fig. 7** shows that for each subject, an increase in activation is observed for higher incongruencies, although the magnitude, shape and slope of this relationship varies between subjects. Specifically, the subject showing the weakest relationship between incongruence and cortical activation (**RV**) was also poor at processing disparity-defined slant. This subject showed a poor correlation between objective and subjective levels of incongruence (**Fig. 2**), as well as a weak correlation between incongruence and alternation rate (**Fig. 3**).

**Figure 8:**
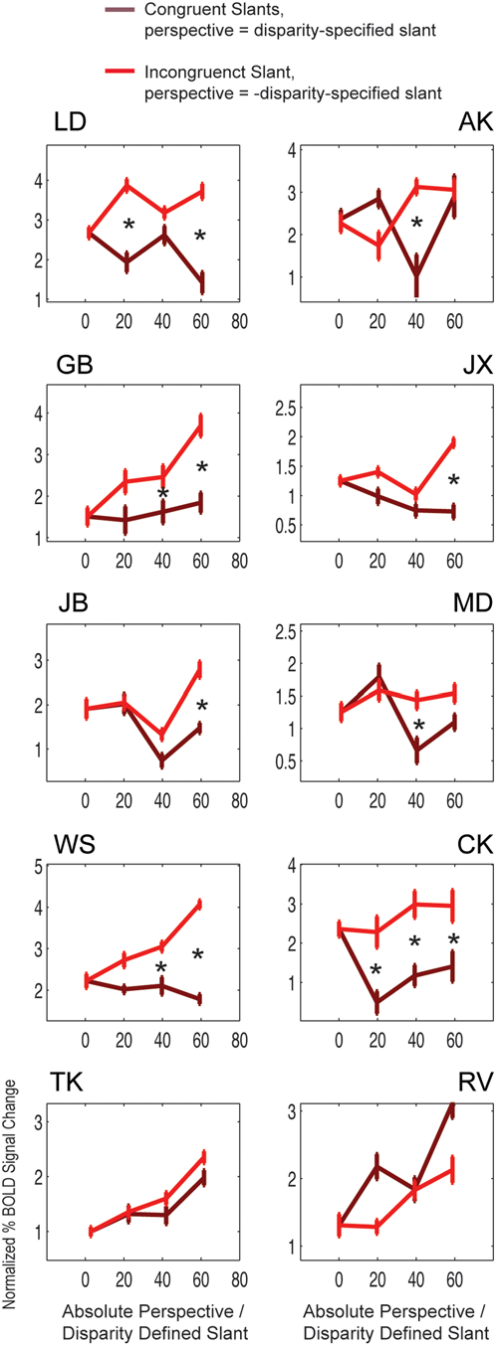
Comparison of activity levels. Comparison of activity levels in the intraparietal sulcus for stimuli containing equal (*no incongruence*) and opposite (*incongruence*) levels of disparity- and perspective-defined slants, plotted for individual subjects. Here, the data is somewhat less reliable, as these graphs are created from a small subset of the total number of stimuli presented. Regardless, a similar trend is observed for most subjects: increasing incongruence is accompanied by increased cortical activation, while increasing slant levels while keeping them equal in sign (no incongruence) did not increase cortical activation. Symbols denote whether a particular comparison (e.g. incongruent slants of 40° versus congruent slants of 40°) was found to be significantly different. The data of subject RV did not show this trend, in accordance with this subject' poor estimation of disparity-defined slants and the weak relationship between cortical activation and incongruence. These data show that activation reflects perceived incongruence, independent of disparity- and perspective-defined slants. Error bars denote SEM.

**Fig. 8** plots the direct comparison between equal and opposite slant stimuli for each individual subject, demonstrating that in most subjects, increases in activation are observed when disparity- and perspective-defined slants increased in opposite directions, compared to when they increased congruently. In summary, activation in the anterior IPS is correlated with incongruence, independent of disparity- and perspective-defined slants.

#### fMRI results: The effect of incongruence in lower visual areas

**Figure 9:**
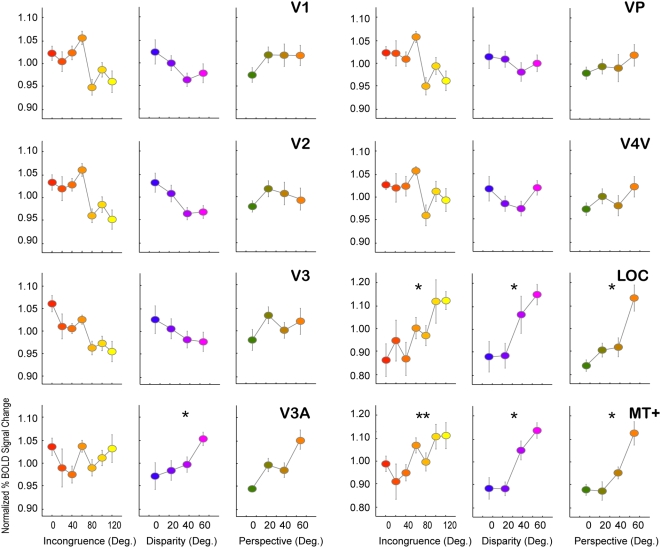
Activation in visual areas. Analysis of the correlations between stimulus measures (from left to right column incongruence, disparity- and perspective-defined slant) and cortical activation in all localized visual areas (V1,V2,V3,V3A, VP and V4V) as well as area MT+ and LOC. Of these areas, only MT+ and LOC show significant increase in activation for higher levels of incongruence. However, the effect of incongruence in these areas cannot be dissociated from the increase in the level of disparity- and perspective-defined slant, as was demonstrated from the activation within aIPS: similar statistical results were obtained for each measure in these areas. Asterisks denote significant fits between data and measure (p<0.05). Error bars denote SEM.

We also investigated the effects of incongruence on cortical responses in retinotopic visual areas V1 through V4V as well as areas LOC and MT+, using a region-of-interest-based analysis (**Fig. 9**). For each individual subject, we identified these visual areas using conventional retinotopic mapping and localization techniques (*see methods*). The effect of increased incongruence, increased absolute disparity, and perspective-defined slants on each of these areas was then ascertained. Interestingly, the activation within early visual areas V1, V2 and V3 seems to decrease for higher levels of disparity-defined slant (although this did not reach significance). This pattern is abruptly reversed in dorsal visual area V3A, which shows positively correlated activity with increased disparity-defined slant. The correlation between activation and increased perspective-defined slant did not reach significance, although it showed a similar relationship. Of all regions of interest, only area LOC (lateral occipital complex) and area MT+ showed an increase in activity for higher levels of incongruence. However, the effect of incongruence could not be dissociated from the increases due to the level of disparity- and perspective-defined slant. A comparison between slant conditions of equal and opposite sign revealed that areas LOC and MT+ showed a positive increase in activity as a function of slant, but activity was no greater for incongruent stimuli than for congruent stimuli that shared the same degree of disparity- and perspective-based slant.

## Discussion

During rivalry between perceptual interpretations of visual slant, we found that the dynamics of bistable perception are influenced by incongruence: increased incongruence between slants led to increased alternation rates. Furthermore, subjective (i.e. perceived) incongruence predicted alternation rates more accurately than objective (i.e. presented) incongruence, suggesting that the dynamics of bistable perception are dependent not on the absolute magnitude of cues or the objective incongruence between cues, but instead depend on the perceived incongruence between these cues. The transformation from objective to subjective incongruence is likely to be a function of how different cues are weighted and combined by subjects.

A neural correlate of these systematic increases in perceptual incongruence was identified bilaterally in the anterior part of IPS and to a lesser extent in areas MT+ and LOC. However, for these two latter areas, activation related to incongruence could not be dissociated from activation related to increasing the absolute perspective- and disparity-defined slant. By presenting stimuli briefly, we focused on activation related to the initial perception (*both sensory and perceptual*) of the stimulus, prior to the onset of alternations. It is commonly believed that bistable perception arises from neural competition between conflicting perceptual signals [1], [36]. However, there has been little systematic investigation of how the brain responds to varying levels of perceptual incongruence prior to entering bistable perceptual states. Here, we show that systematic increases in response strength as a function of perceptual incongruence can be found in anterior IPS, an area implicated in the processing of depth [37]–[42]. An fMRI study of disparity processing in both humans and monkeys found that a region in the posterior (or caudal) intraparietal sulcus was responsive to stereoscopic depth in both species [43]. Single-unit recordings in macaque caudal intraparietal sulcus (CIP) have revealed neurons that are sensitive to surface orientation based on cues such as perspective, texture and disparity [40]–[42]. Moreover, the majority of CIP neurons are biased towards disparity-defined surfaces, showing greater sensitivity to disparity-defined slants as compared to perspective- or texture-defined slants. Compared to these regions of interest, the activation found in the present study is located more anteriorly along the IPS. Previous studies have shown that the anterior IPS is activated when subjects perform a surface orientation discrimination task that requires discriminating between surfaces defined by texture, perspective and disparity [38]. This suggests that both posterior IPS (homologue to macaque CIP) and anterior IPS are both part of the dorsal stream of visual processing, which is responsible for integrating depth cues to represent the spatial organization of the local environment and for guiding actions within that environment. The sensitivity of the intraparietal sulcus to the degree of perceptual incongruence suggests that it may have an important role in signaling the need to reinterpret potentially ambiguous depth cues contained in the visual scene, thereby initiating bistable perception.

In our stimulus, incongruence arose from conflicting depth cues, while in other forms of visual bistability it arises, for example, from half-images that cannot be binocularly fused (i.e. binocular rivalry). As an alternative explanation, the activity within the intraparietal sulcus might signal perceptual incongruence, regardless of its (sub)modality (e.g. depth). Indeed there is a growing literature that suggests a role for the parietal cortex (particularly the IPS) in binding visual, auditory, and tactile information (review in [44]).

It has been suggested that the prefrontal cortex might be involved in initiating perceptual alternations under conditions of bistability [8], [9]; might these same regions have a role in detecting perceptual incongruence? We found some suggestion of prefrontal regions showing increased activity as a function of the degree of incongruence, but these effects did not reach significance. In a previous study of slant rivalry, we also found no reliable evidence of increased activity in prefrontal areas during spontaneous perceptual alternations [19]. In our view, the precise functional role of the prefrontal cortex in bistable perception remains unclear. A previous fMRI study showed evidence of covariation of activity between regions of the prefrontal cortex and early visual areas during binocular rivalry [9]. Parallel studies using magnetoencephalography (MEG) have reported widespread intra- and inter-hemispheric synchronized activity during binocular rivalry [45], with evidence of these dynamic networks extending from early visual areas to higher order areas of the parietal and frontal lobe. However, a recent study found that the differences in frontal activity between two perceptual states might be explained by differences in observer biases for those two perceptual states [46]. Concerning the frontal MEG activity that is thought to accompany visual rivalry [45], a recent study reported that measures of coherence between different sensors may be dominated by signals from a common occipital source [47], suggesting that previous claims about widespread synchronized networks during binocular rivalry are premature.

We found that activity in area V3A increased with greater disparity-specified slants, consistent with the proposed role of this region in stereoscopic depth perception [48]–[54]. In previous work, exploiting the benefit of using the slant-rivalry stimulus to dissociate between sensory processing of disparity and the sensation of stereopsis, we have found a clear correlation between the activity in V3A and perceptual alternations towards a disparity-dominated percept [19]. Collectively, these findings suggest that V3A reflects a relatively early stage of visual processing involved in extracting the disparity-defined slant of surfaces.

Area LOC showed increases in activation for higher levels of incongruence. However this effect could not be dissociated from activation resulting from changes in the level of disparity- and perspective-defined slant. Area LOC is commonly associated with the processing of visual shape [25] and is sensitive to shapes defined by many visual cues [26], including disparity [27]. There is further evidence that the ventral route of visual processing relies on multiple depth cues to determine shape. For example, some V4 neurons show strong tuning to orientation in the third dimension conveyed by disparity cues [55]. Inferior temporal (IT) neurons in the macaque monkey show selectivity for disparity-defined shapes [56]–[58]. In addition, some of these neurons also show selectivity to texture-defined slants, consistent with their proposed role in representing 3D shape [59]. Of particular relevance to the present study, it has been found that LOC is able to integrate different depth cues (*e.g. perspective and disparity*) to extract 3D information from objects [60].

Area MT+ showed a similar pattern of results as was found for area LOC; activity increased with to increasing levels of incongruence, but this effect could not be dissociated from changes in the levels of perspective and disparity-defined slant. Although MT+ is typically considered an area involved in processing motion signals, macaque MT does contain disparity-sensitive cells, which show a topographic organization similar to direction tuning. Furthermore, MT cells appear to be tuned to disparity-defined slants [61]. Nevertheless, the shape selectivity in MT makes it an unlikely candidate to be involved in the process needed to resolve the ambiguity and the level of this ambiguity between slant-specifying cues.

What type of neural mechanism might underlie both the increased alternation rate and the increased activation found at higher incongruencies? Although there is much debate about what exactly competes during bistable perception, it is generally assumed that some form of neural competition must underlie it. The competition model is a suitable candidate to account for the positive correlation we find between alternation rate and incongruence. We suggest that when representations are quite similar (low incongruence), neural populations coding these representations (partially) overlap. Increasing incongruence leads to a decrease in overlap between neural representations, leading to increased competition. This increased competition could account for the higher alternation rates. Also, the decrease in overlap would involve more and more neurons being recruited to represent the two percepts as they become increasingly distinct, thereby increasing the net activity across both neuronal populations. Alternatively, the increase in dissimilarity increases the strength of mutual inhibition between both neural populations. In both cases, higher incongruencies will lead to a higher metabolic demand of these neurons, leading to a higher BOLD signal in fMRI, providing a possible explanation for our results that show increased incongruence increases cortical activation.

In conclusion, we used a novel approach to study the mechanisms of depth perception and perceptual bistability by combining psychophysics and functional imaging. Our aim was to investigate how the brain responds to increasingly incongruent information. This allowed us to identify the influence of parametric changes in incongruence on both perceptual processes and the cortical mechanisms that underlie these processes. Our results demonstrate that alternation rate increases with higher incongruencies for slant rivalry. Interestingly, perceived (subjective) incongruence predicted alternation rate more accurately than presented (objective) incongruence. We found increased activation within the anterior part of the intraparietal sulcus (IPS) at higher incongruencies. These effects were present even prior to the onset of perceptual alternations, as we presented the stimuli too briefly for alternations to occur and shape the pattern of activity we observed. This suggests that this area is important for assessing the level of incongruence between perceptual states. Possibly, the activation of these areas in response to a specific perceived incongruence may influence or determine the frequency of subsequent perceptual alternations. We account for increased alternation rate and activation by assuming that perceptual representations become more distinct and less overlapping at higher incongruencies, thereby recruiting more neurons for the competitive process in bistability. To summarize, we demonstrate that the brain takes into account the relative difference between interpretations in its attempt to reconcile these into a coherent and stable perceptual experience and that evidence of this reconciliation process can be seen in both the temporal dynamics of bistability and in cortical responses prior to the onset of bistability.

## References

[1] Blake R, Logothetis NK (2002). Visual Competition.. Nat Rev Neurosci.

[2] Hol K, Koene A, Van Ee R (2003). Attention-biased multi-stable surface perception in three-dimensional structure-from-motion.. Journal of Vision.

[3] Suzuki S, Peterson MA (2000). Multiplicative effects of intention on the perception of bistable apparent motion.. Psychological Science.

[4] Pettigrew JD, Miller SM (1998). A sticky interhemispheric switch in bipolar disorder.. Proc R Soc London Ser B.

[5] Tononi G, Edelman GM (2000). Schizophrenia and the mechanisms of conscious integration.. Brain Res Brain Res Rev.

[6] Logothetis NK, Schall JD (1989). Neuronal correlates of subjective visual perception.. Science.

[7] Leopold DA, Logothetis NK (1996). Activity changes in early visual cortex reflects monkey' precepts during binocular rivalry.. Nature.

[8] Lumer ED, Friston KJ, Rees G (1998). Neural correlates of perceptual rivalry in the human brain.. Science.

[9] Lumer ED, Rees G (1999). Covariation of activity in visual and prefrontal cortex associated with subjective visual perception.. Proc Natl Acad Sci USA.

[10] Kleinschmidt A, Bushel C, Zeki S, Frackowiak RS (1998). Human brain activity during spontaneously reversing perception of ambiguous figures.. Proc R Soc Lond B.

[11] Tong F, Nakayama K, Vaughan JT, Kanwisher N (1998). Binocular rivalry and visual awareness in human extrastriate cortex.. Neuron.

[12] Muckli L, Kriegeskorte N, Lanfermann H, Zanella FE, Singer W (2002). Apparent motion: event-related functional magnetic resonance imaging of perceptual switches and states.. J Neurosci.

[13] Haynes JD, Rees G (2005). Predicting the stream of consciousness from activity in human visual cortex.. Curr Biol.

[14] Slotnick SD, Yantis S (2005). Common neural substrates for the control and effects of visual attention and perceptual bistability.. Brain Res Cogn Brain Res.

[15] Sterzer P, Kleinschmidt A (2006). A neural basis for inference in perceptual ambiguity.. Proc Natl Acad Sci USA.

[16] van Ee R, van Dam LCJ, Erkelens CJ (2002). Bi-stability in perceived slant when binocular disparity and monocular perspective specify different slants.. J Vis.

[17] van Ee R (2005). Dynamics of perceptual bi-stability for stereoscopic slant rivalry and a comparison with grating, house-face, and Necker cube rivalry.. Vis Res.

[18] Knapen THJ, van Ee R (2006). Slant perception, and its voluntary control, do not govern the slant aftereffect: multiple slant signals adapt independently.. Vision Research.

[19] Brouwer GJ, van Ee R, Schwarzbach J (2005). Activation in visual cortex correlates with the awareness of stereoscopic depth.. J Neurosci.

[20] van Ee R, Richards W (2002). A planar and a volumetric test for stereoanomaly.. Perception.

[21] Brouwer GJ, van Ee R (2007). Visual cortex allows prediction of perceptual states during ambiguous structure-from-motion.. J Neurosci.

[22] Tootell RB, Mendola JD, Hadjikhani NK, Ledden PJ, Liu AK (1997). Functional analysis of V3A and related areas in human visual cortex.. J Neurosci.

[23] DeYoe EA, Carman GJ, Bandettini P, Glickman S, Wieser J (1996). Mapping striate and extrastriate visual areas in human cerebral cortex.. Proc Natl Acad Sci USA.

[24] Tootell RB, Hadjikhani NK, Vanduffel W, Liu AK, Mendola JD (1998a). Functional analysis of primary visual cortex (V1) in humans.. Proc Natl Acad Sci USA.

[25] Malach R, Reppas JB, Benson RR, Kwong KK, Jiang H (1995). Object-related activity revealed by functional magnetic resonance imaging in human occipital cortex.. Proc Natl Acad Sci USA.

[26] Grill-Spector K, Kushnir T, Hendler T, Edelman S, Itzchak Y (1998). A sequence of object-processing stages revealed by fMRI in the human occipital lobe.. Hum Brain Mapp.

[27] Kurtz Z, Kanwisher N (2000). Cortical regions involved in perceiving object shape.. J Neurosci.

[28] van Dam LC, van Ee R (2005). The role of (micro)saccades and blinks in perceptual bi-stability from slant rivalry.. Vision Res.

[29] Kriegeskorte N, Goebel R (2001). An efficient algorithm for topologically correct segmentation of the cortical sheet in anatomical MR volumes.. NeuroImage.

[30] Wandell BA (2000). Computational neuroimaging of human visual cortex.. Annu Rev Neurosci.

[31] Slotnick SD, Yantis S (2003). Efficient acquisition of human retinotopic maps.. Hum Brain Map.

[32] Talairach J, Tournoux P (1988). Co-planar stereotaxic atlas of the human brain.

[33] Boynton GM, Engel SA, Glover GH, Heeger DJ (1996). Linear systems analysis of functional magnetic resonance imaging in human V1.. J Neurosci.

[34] Friston KJ, Holmes AP, Worsley KJ, Poline J.-P, Frith CD (1995). Statistical parametric maps in functional imaging: a general linear approach.. Hum Brain Map.

[35] van Ee R, Adams WJ, Mamassian P (2003). Bayesian modeling of cue interaction: bi-stability in stereoscopic slant perception.. J Optical Society of America A.

[36] Leopold DA, Logothetis NK (1999). Multistable phenomena: changing views in perception.. Trends Cog Sci.

[37] Shikata E, Tanaka Y, Nakamura H, Taira M, Sakata H (1996). Selectivity of the parietal visual neurons in 3D orientation of surface of stereoscopic stimuli.. Neuroreport.

[38] Shikata E, Hamzei F, Glauche V, Knab R, Dettmers C (2001). Surface orientation discrimination activates caudal and anterior intraparietal sulcus in humans: an event-related fMRI study.. J Neurophysiol.

[39] Shikata E, Hamzei F, Glauche V, Koch M, Weiler C (2003). Functional properties and interaction of the anterior and posterior intraparietal areas in humans, an event-related fMRI study.. Eur J Neurosci.

[40] Tsutsui K, Jiang M, Yara K, Sakata H, Taira M (2001). Integration of perspective and disparity cues in surface-orientation-selective neurons of area CIP.. J Neurophysiol.

[41] Tsutsui K, Sakata H, Naganuma T, Taira M (2002). Neural correlates for perception of 3D surface orientation from texture gradient.. Science.

[42] Taira M, Tsutsui K, Jiang M, Yara K, Sakata H (2000). Parietal neurons represent surface orientation form the gradient of binocular disparity.. J Neurophysiol.

[43] Tsao DY, Vanduffel W, Sasaki Y, Fize D, Knutsen TA (2003). Stereopsis activates V3a and caudal intraparietal areas in macaques and humans.. Neuron.

[44] Cusack L (2005). The intraparietal sulcus and perceptual organization.. Journal of Cognitive Neuroscience.

[45] Tononi G, Srinivasan R, Russell DP, Edelman GM (1998). Investigating neural correlates of conscious perception by frequency-tagged neuromagnetic responses.. PNAS.

[46] Raemaekers M, van der Schaaf ME, van Ee R, van Wezel RJA (2009). Widespread fMRI activity differences between perceptual states in rivalry are correlated with differences in observer biases.. Brain Research.

[47] Kamphuisen AP, Bauer M, van Ee R (2008). No evidence for widespread synchronized networks in binocular rivalry: Meg frequency tagging entrains primarily early visual cortex.. Journal of Vision.

[48] Backus BT, Fleet DJ, Parker AJ, Heeger DJ (2001). Human cortical activity correlates with stereoscopic depth perception.. J Neurophysiol.

[49] Neri P, Bridge H, Heeger DJ (2004). Stereoscopic processing of absolute and relative disparity in human visual cortex.. J Neurophysiol.

[50] Kourtzi Z, Erb M, Grodd W, Bulthoff HH (2003). Representation of the perceived 3-D object shape in the human lateral occipit*al complex*.. Cereb Cortex.

[51] Hinkle DA, Connor CE (2002). Three-dimensional orientation tuning in macaque area V4.. Nat Neurosci.

[52] Chandrasekaran C, Canon V, Dahmen CJ, Kourtzi Z, Welchman AE (2007). Neural correlates of disparity-defined shape discrimination in the human brain.. Journal of Neurophysiology.

[53] Georgieva S, Peeters R, Kolster H, Todd JT, Orban GA (2009). The processing of three-dimensional shape from disparity in the human brain.. Journal of Neuroscience.

[54] Preston TJ, Li S, Kourtzi Z, Welchman AE (2008). Multivoxel pattern selectivity for perceptually relevant binocular disparities in the human brain.. Journal of Neuroscience.

[55] Janssen P, Vogels R, Orban GA (1999). Macaque inferior temporal neurons are selective for disparity-defined three-dimensional shapes.. Proc Natl Acad Sci USA.

[56] Janssen P, Vogels R, Orban GA (2000). Three-dimensional shape coding in inferior temporal cortex.. Neuron.

[57] Janssen P, Vogels R, Orban GA (2000). Selectivity for 3D shape that reveals distinct area within macaque inferior temporal cortex.. Science.

[58] Janssen P, Vogels R, Orban GA (2001). Macaque inferior temporal neurons are selective for three-dimensional boundaries and surfaces.. J Neurosci.

[59] Lui Y, Vogels R, Orban GA (2004). Convergence of depth from texture and depth from disparity in macaque inferior temporal cortex.. J Neurosci.

[60] Welchman AE, Deubelius A, Conrad V, Bulthoff HH, Kourtzi Z (2005). 3D Shape perception from combined depth cues in human visual cortex.. Nat Neurosci.

[61] Nguyenkim JD, DeAngelis GC (2003). Disparity-based coding of three-dimensional surface orientation by macaque middle temporal neurons.. J Neurosci.

[62] Adams WJ, Banks MS, van Ee R (2001). Adaptation to three-dimensional distortions in human vision.. Nature Neuroscience.

[63] van Ee R, Anderson BL (2001). Motion direction, speed, and orientation in binocular matching.. Nature.

